# Novel Influenza A(H7N2) Virus in Chickens, Jilin Province, China, 2014

**DOI:** 10.3201/eid2010.140869

**Published:** 2014-10

**Authors:** Jianzhong Shi, Guohua Deng, Xianying Zeng, Huihui Kong, Xiaoyu Wang, Kunpeng Lu, Xiurong Wang, Guodong Mu, Xiaolong Xu, Pengfei Cui, Hongmei Bao, Guobin Tian, Hualan Chen

**Affiliations:** State Key Laboratory of Veterinary Biotechnology–Harbin Veterinary Research Institute, Harbin, China (J. Shi, G. Deng, X. Zeng, H. Kong, K. Lu, X. Wang, X. Xu, P. Cui, H. Bao, G. Tian, H. Chen);; Jilin Province Center for Animal Disease Control and Prevention, Changchun, China (X. Wang, G. Mu)

**Keywords:** influenza virus, H7N2 reassortant, H7N9, H9N2, influenza, viruses, China, chickens

## Abstract

In February 2014, while investigating the source of a human infection with influenza A(H7N9) virus in northern China, we isolated subtypes H7N2 and H9N2 viruses from chickens on the patient’s farm. Sequence analysis revealed that the H7N2 virus is a novel reassortant of H7N9 and H9N2 viruses. Continued surveillance is needed.

Influenza subtype H7 viruses have been detected in poultry worldwide; associated human disease ranges from mild to severe (*1*–*8*). Human infections with influenza A(H7N9) viruses were first identified in China in March 2013 (*9*). As of March 11, 2014, a total of 375 laboratory-confirmed cases of human infection with influenza A(H7N9) virus, including 115 deaths, had been reported to the World Health Organization (*10*).

On February 21, 2014, the National Health and Family Planning Commission of China notified the World Health Organization of a laboratory-confirmed case of human infection with influenza virus subtype H7N9 (*11*). The patient was a 50-year-old farmer who lived in Jilin Province and traded chickens for a living. He became ill on February 15 and was confirmed to be infected with H7N9 virus on February 21. He recovered 2 weeks later. Although H7N9 viruses had been detected in live poultry markets in 12 provinces in China (*12*,*13*), the virus had not been detected in Jilin Province, in poultry or humans. To locate the origin of the infection, we conducted influenza virus surveillance among poultry in the patient’s village.

## The Study

Cloacal and tracheal swab samples and serum were collected from 60 of 500 chickens on the patient’s family farm and from 50 chickens in the backyards of 5 neighbors within 3 kilometers. In addition, 36 fecal samples from chickens on the patient’s and neighboring farms were also collected. Each swab or fecal sample was placed in 2 mL of minimal essential medium supplemented with penicillin (2,000 U/mL) and streptomycin (2,000 U/mL). Virus was isolated by using 10-day-old specific pathogen–free embryonated chicken eggs. Hemagglutinin (HA) and neuraminidase (NA) subtypes were determined as described previously (*14*). Hemagglutination inhibition assay with 0.5% chicken erythrocytes was used to test for antibodies against H7 virus in the chicken serum samples.

From the cloacal swab samples from the patient’s farm, 3 viruses were isolated (a Newcastle disease virus, an H9N2 influenza virus, and an H7N2 influenza virus); virus was not isolated from any sample collected from chickens on the neighboring farms. We designated the influenza viruses as A/chicken/Jilin/SD001/2014(H9N2) and A/chicken/Jilin/SD020/2014(H7N2).

We then fully sequenced the genomes of A/chicken/Jilin/SD020/2014(H7N2) and A/chicken/Jilin/SD001/2014(H9N2) (GenBank accession nos. KM054788–KM054803) and found that the NA and nonstructural (NS) genes of A/chicken/Jilin/SD020/2014(H7N2) are similar to those of A/chicken/Jilin/SD001/2014(H9N2); identities were 99.1% and 100%, respectively. The other 6 genes were closely related to those of the H7N9 viruses that had been isolated from poultry or humans during 2013–2014 in China; identities were 99.5%–99.9% (Table). In the phylogenetic trees, the HA of A/chicken/Jilin/SD020/2014(H7N2) clustered with that of the recently emerged H7N9 viruses (Technical Appendix, Figure, panel A), whereas, the NA, polymerase basic (PB) 2, PB1, polymerase acidic (PA), nucleocapsid protein (NP), and NS genes of A/chicken/Jilin/SD020/2014(H7N2) and A/chicken/Jilin/SD001/2014(H9N2) clustered with those of the H9N2 viruses (Technical Appendix Figure, panels B–F, H). However, the matrix (M) gene of the 2 viruses remained on different forks; the M gene of A/chicken/Jilin/SD020/2014(H7N2) clustered with the H7N9 or H9N2 viruses, and the M gene of A/chicken/Jilin/SD001/2014(H9N2) clustered with the viruses from other subtypes (Technical Appendix Figure, panel G). These results indicate that A/chicken/Jilin/SD020/2014(H7N2) is a novel reassortant of H7N9 and H9N2 viruses. With the approval of the Review Board of Harbin Veterinary Research Institute, we tested the virulence of the A/chicken/Jilin/SD020/2014(H7N2) in animals in Biosafety Level 3 laboratories.

**Table:** Homology among influenza viruses closely related to avian influenza virus A/chicken/Jilin/SD020/2014(H7N2) from Jilin, China, 2014*

Gene	Virus	Homology, %
HA	A/chicken/Zhejiang/S4135/2013(H7N9)	99.6
NA	A/chicken/Jilin/SD001/2014(H9N2)	99.1
PB2	A/chicken/Zhejiang/S4135/2013(H7N9)	99.9
PB1	A/chicken/Zhejiang/S4135/2013(H7N9)	99.5
PA	A/chicken/Hunan/SD015/2014(H7N9)	99.7
NP	A/Shanghai/02/2013(H7N9)	99.8
M	A/Shanghai/5190T/2013(H7N9)	99.7
NS	A/chicken/Jilin/SD001/2014(H9N2)	100

*HA, hemagglutinin; NA, neuraminidase; NP, nucleoprotein; M, matrix; NS, nonstructural; PA, polymerase basic; PB, polymerase basic.

Similar to the H7N9 viruses (*12*,*13*), A/chicken/Jilin/SD020/2014(H7N2) also has the single basic amino acid arginine in its HA cleavage site. We determined the intravenous pathogenicity index of A/chicken/Jilin/SD020/2014(H7N2) as described previously (*13*). None of the chickens showed signs of disease or died during the 10-day observation period; the intravenous pathogenicity index was 0, indicating that this H7N2 virus in chickens is nonpathogenic.

**Figure 1:**
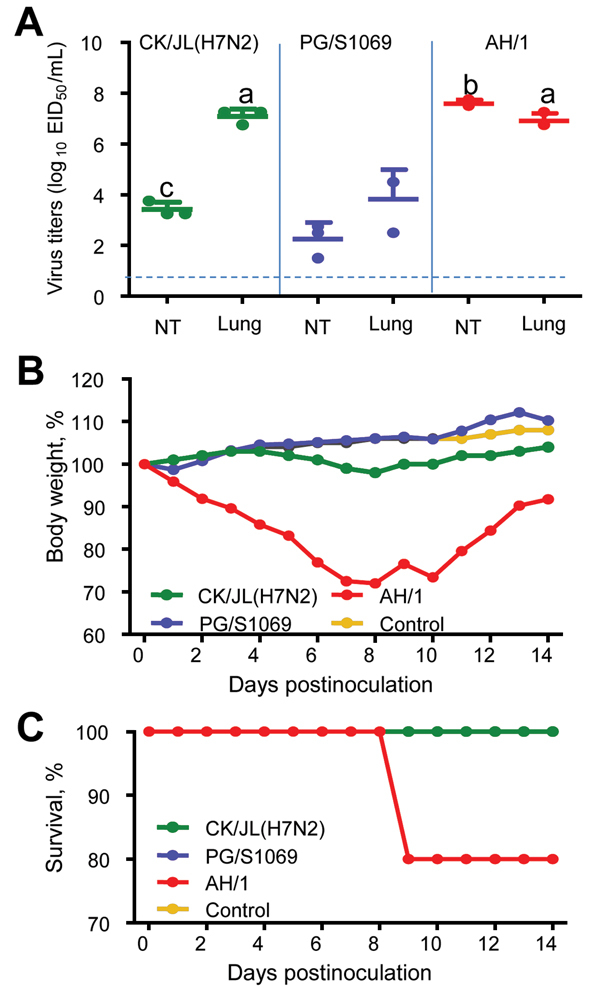
Replication and virulence of avian influenza A virus subtypes H7N2 and H7N9 in mice. A) Virus titers in organs of mice. The data shown are the mean ± SD for each group. Because virus was not detected from spleen, kidney, or brain of any mouse, data for these organs are not shown. a, p<0.01 compared with the corresponding value for the A/pigeon/Shanghai/S1069/2013-inoculated group; b, p<0.01 compared with the corresponding value for the A/chicken/Jilin/SD020/2014(H7N2)-inoculated and A/pigeon/Shanghai/S1069/2013-inoculated groups; c, p<0.05 compared with the corresponding value for the A/pigeon/Shanghai/S1069/2013-inoculated group. The dashed line indicates the lower limit of detection. NT, nasal turbinates. B) Percentages of body weight changes of mice. C) Percentages of mice that survived. CK/JL (H7N2), A/chicken/Jilin/SD020/2014(H7N2); PG/S 1069, A/pigeon/Shanghai/S1069/2013; AH/1, A/Anhui/1/2013; EID_50_, 50% egg infectious dose.

We inoculated groups of eight 6-week-old female BALB/c mice with 10^6^ 50% egg infectious doses of A/chicken/Jilin/SD020/2014(H7N2) and 2 H7N9 viruses, A/pigeon/Shanghai/S1069/2013, and A/Anhui/1/2013. On day 3 postinfection, 3 mice in each group were killed and their organs (nasal turbinates, lungs, spleens, kidneys, and brains) were collected for virus titration. For 14 days, the remaining 5 mice were observed for body weight changes and survival. In the mice, replication of all 3 viruses was detected in the nasal turbinates and lungs but not in other organs (Figure 1, panel A); the titers in lungs of mice infected with H7N2 virus were comparable to those in the lungs of mice infected with the human H7N9 virus A/Anhui/1/2013 and were significantly higher than those in the lungs of mice infected with the avian H7N9 virus A/pigeon/Shanghai/S1069/2013 (Figure 1, panel A). The A/Anhui/1/2013 virus–infected mice showed up to a 30% loss of body weight, and 1 mouse died during the observation period (Figure 1, panels B and C). Although none of the mice infected with the H7N2 virus or A/pigeon/Shanghai/S1069/2013 virus died during the observation period, loss of body weight was slightly more for the H7N2-infected mice than for the A/pigeon/Shanghai/S1069/2013-infected mice and the control mice (Figure 1, panel B).

**Figure 2:**
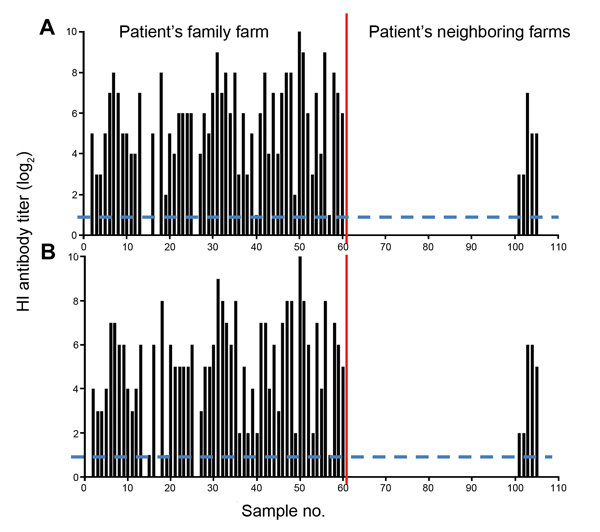
Antibody titers in chicken serum against H7 influenza viruses. The hemagglutination inhibition (HI) antibody titers of the serum against the H7N2 virus A/chicken/Jilin/SD020/2014(H7N2) (A) and the H7N9 virus A/pigeon/Shanghai/S1069/2013 (B) were determined with 0.5% (vol/vol) chicken erythrocytes. Each bar represents the hemagglutination inhibition antibody value of an individual serum sample**.** Dashed lines indicate limit of detection.

We also investigated antibody responses in serum samples from chickens in the patient’s village. In 55 of the 60 serum samples collected from the chickens on the patient’s family farm, hemagglutination inhibition assay indicated that antibody titers against H7N2 or H7N9 virus ranged from 2 to 1,024. Only 5 of the 50 serum samples collected from the chickens on the patient’s neighboring farms had hemagglutination inhibition antibody titers, which ranged from 2 to 128 (Figure 2). 

## Conclusions

The H7N2 influenza virus isolated from a chicken in Jilin Province in northern China was a novel reassortant that derived its HA, PB2, PB1, PA, NP, and M genes from the H7N9 virus that emerged in China in 2013. Although we did not find any H7N9 viruses in chickens during this investigation, the fact that the owner of the chickens was infected with an H7N9 virus indicates that H7N9 viruses might have circulated among these chickens. The NA and NS genes of the novel H7N2 virus are closely related to those of the H9N2 virus that was isolated from chickens on the same farm, providing direct evidence that H7N9 viruses continue to evolve and reassort with H9N2 viruses in poultry in China.

H7N9 isolates from humans replicate much more efficiently and are more lethal in mice than are H7N9 isolates from birds (*13*). The mutation of glutamic acid to lysine at position 627(E627K) in PB2 contributes to this difference (*15*). The H7N2 virus does not have the PB2 627K mutation, but its replication in the lungs of mice is comparable to that of human H7N9 virus and significantly higher than that of avian H7N9 virus. These findings suggest that the continued circulation of H7 viruses in nature will enable them to acquire more mutations or new gene constellations that might increase their virulence in animals or humans.

The nonpathogenic nature of H7 viruses in poultry enables them to replicate silently in birds. The high positive ratio of antibody against H7 viruses detected by hemagglutination assay and the huge diversity of antibody levels among chickens from the H7N9 patient’s farm demonstrate that the H7 viruses might have been introduced and circulated in these birds for several weeks before they were detected. These findings highlight the challenges of trying to eradicate low pathogenicity influenza subtype H7 viruses from nature and the need for continued surveillance and monitoring of H7 viruses in poultry in China.

Technical Appendix Phylogenetic trees of avian influenza A(H7N2) virus. The tree topology was evaluated by 1,000-bootstrap analyses. The trees of H7 hemagglutinin (A), polymerase basic 2 (C), polymerase basic 1 (D), polymerase acidic (E), nucleocapsid (F), matrix (G), and nonstructural (H) were rooted to A/EQ/Prague/1/56 (H7N7), and the N2 neuraminidase tree (B) was rooted to A/duck/Hokkaido/95/01(H2N2). Viruses characterized in the present study are shown in red (H7N2) and pink (H9N2); the recent H7N9 viruses are shown in blue. BP, Baer’s pochard duck; BWT, blue-winged teal; CK, chicken; DK, duck; ENV, environment; EQ, equine; ML, mallard; PG, pigeon; WB, wild bird; WD, wild duck. Scale bars indicate nucleotide substitutions per site.
