# Supplementary figures and images for: Novel Influenza A(H7N2) Virus in Chickens, Jilin Province, China, 2014
Source: Emerg Infect Dis. 2014 Oct;20(10):1719–22. doi: 10.3201/eid2010.140869 (PMC4193184; doi:10.3201/eid2010.140869)

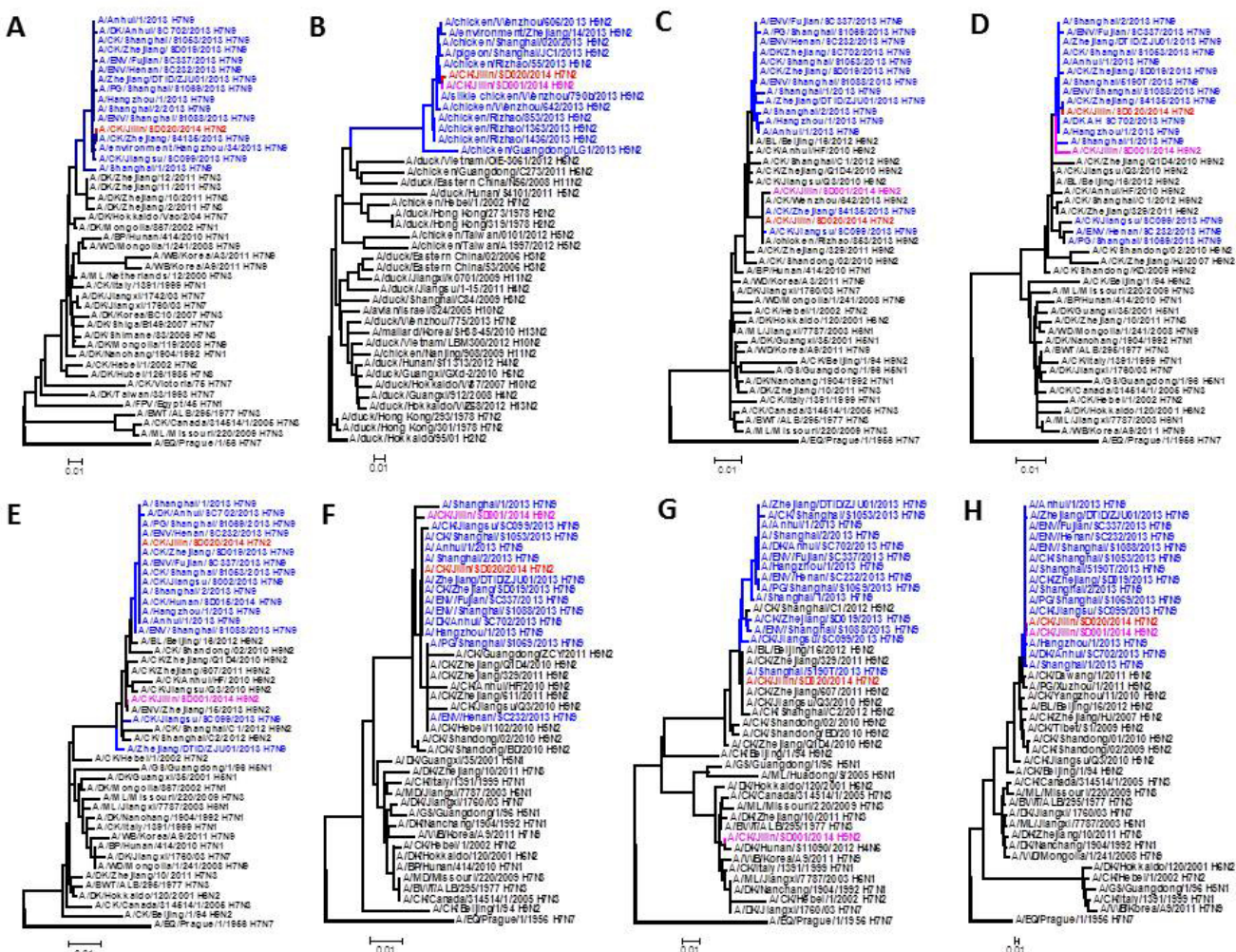

Supplement: Technical Appendix — Phylogenetic trees of avian influenza A(H7N2) virus. The tree topology was evaluated by 1,000-bootstrap analyses. The trees of H7 hemagglutinin (A), polymerase basic 2 (C), polymerase basic 1 (D), polymerase acidic (E), nucleocapsid (F), matrix (G), and nonstructural (H) were rooted to A/EQ/Prague/1/56 (H7N7), and the N2 neuraminidase tree (B) was rooted to A/duck/Hokkaido/95/01(H2N2). Viruses characterized in the present study are shown in red (H7N2) and pink (H9N2); the recent H7N9 viruses are shown in blue. BP, Baer’s pochard duck; BWT, blue-winged teal; CK, chicken; DK, duck; ENV, environment; EQ, equine; ML, mallard; PG, pigeon; WB, wild bird; WD, wild duck. Scale bars indicate nucleotide substitutions per site. [file 14-0869-Techapp-s1.pdf]
